# Supplementary material for: MYL3 protects chondrocytes from senescence by inhibiting clathrin-mediated endocytosis and activating of Notch signaling
Source: Nat Commun. 2023 Oct 4;14:6190. doi: 10.1038/s41467-023-41858-7 (PMC10550997; doi:10.1038/s41467-023-41858-7)

MYL3 protects chondrocytes from senescence by inhibiting clathrin-mediated endocytosis and activating of Notch signaling

Supplementary Figures

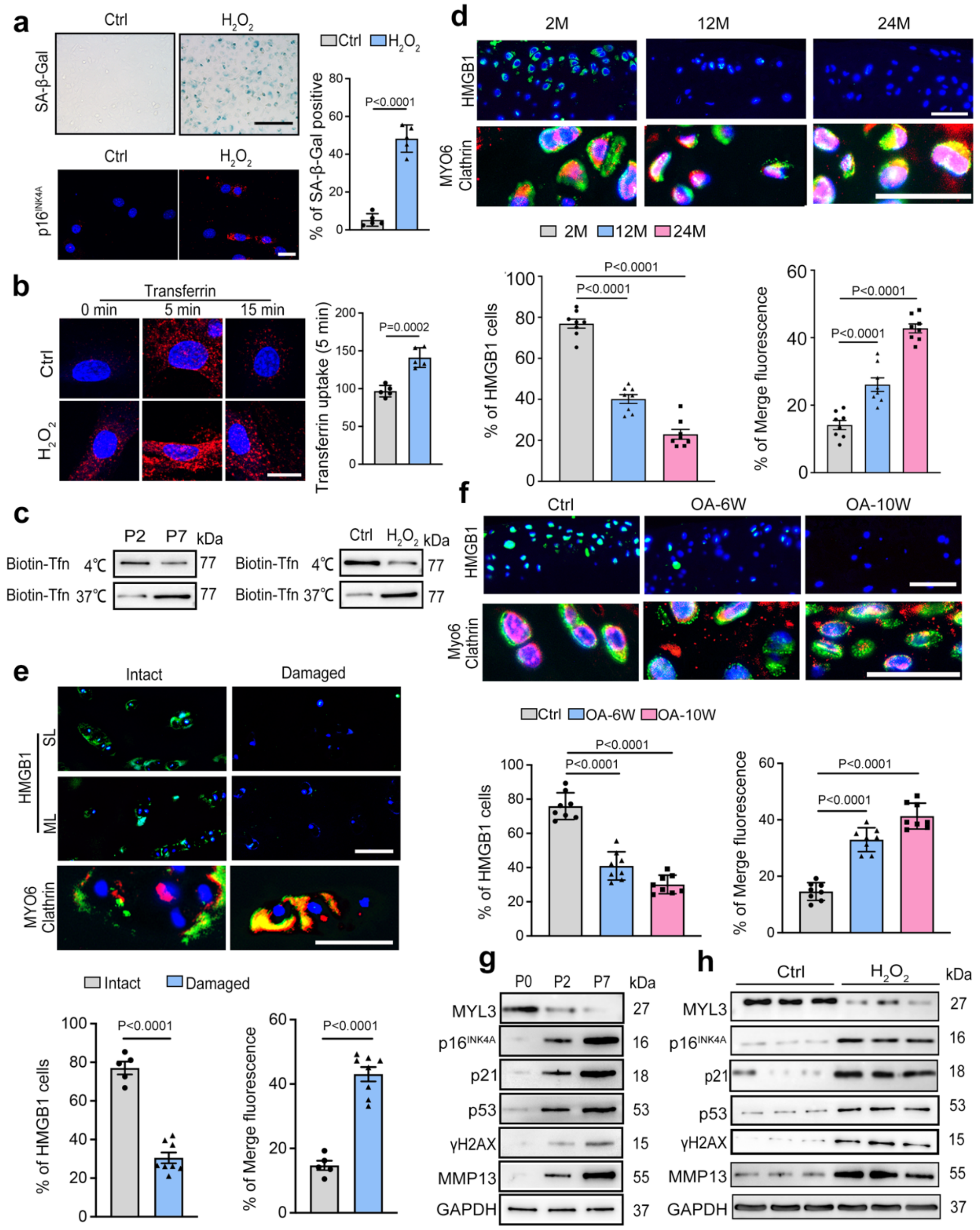

Supplementary Figure 1: CME is enhanced and MYL3 expression is decreased in senescent chondrocytes.

(a) Images and quantification of SA-β-Gal positivity and IF staining of p16^{INK4A} in mouse primary chondrocytes with or without H_2O_2 treatment. n = 5, bars= 25 μm. (b) Images and quantification of transferrin endocytosis in mouse primary chondrocytes with or without H_2O_2 treatment. n = 5, bars= 25 μm. (c) Internalized Biotin-transferrin at passage two or seven and internalized Biotin-transferrin with or without H_2O_2 treatment. (d) Images and quantification of IF staining of HMGB1 and double IF staining of Clathrin and MYO6 in articular cartilage from 2-, 12- and 24-month-old mice, n = 8, bars= 50 μm. (e) Images and quantification of IF staining of HMGB1 and double IF staining of Clathrin and MYO6 in articular cartilage in intact (n = 5) and damaged (n = 9) articular cartilage sections collected from OA patients. bars= 50 μm. (f) Images and quantification of IF staining of HMGB1 and double IF staining of Clathrin and MYO6 in articular cartilage from mice of control, 6- and 10- weeks post DMM surgery. n = 8, bars= 50 μm. (g) Protein levels of MYL3, p16^{INK4A}, p21, p53, γH2AX and MMP13 in primary chondrocytes from control mice at passage 0, 2 and 7. (h) Protein levels of MYL3, p16^{INK4A}, p21 p53 and γH2AX in primary chondrocytes from control mice with or without H_2O_2 treatment. Data are representative of three independent experiments and are all shown as means ± SD. P values are from two-tailed unpaired t-test (a, b, e) and one-way ANOVA test followed by Tukey's post hoc test (d, f). n indicates the number of biologically independent samples, mice per group, or human specimens. Source data are provided as a Source Data file.

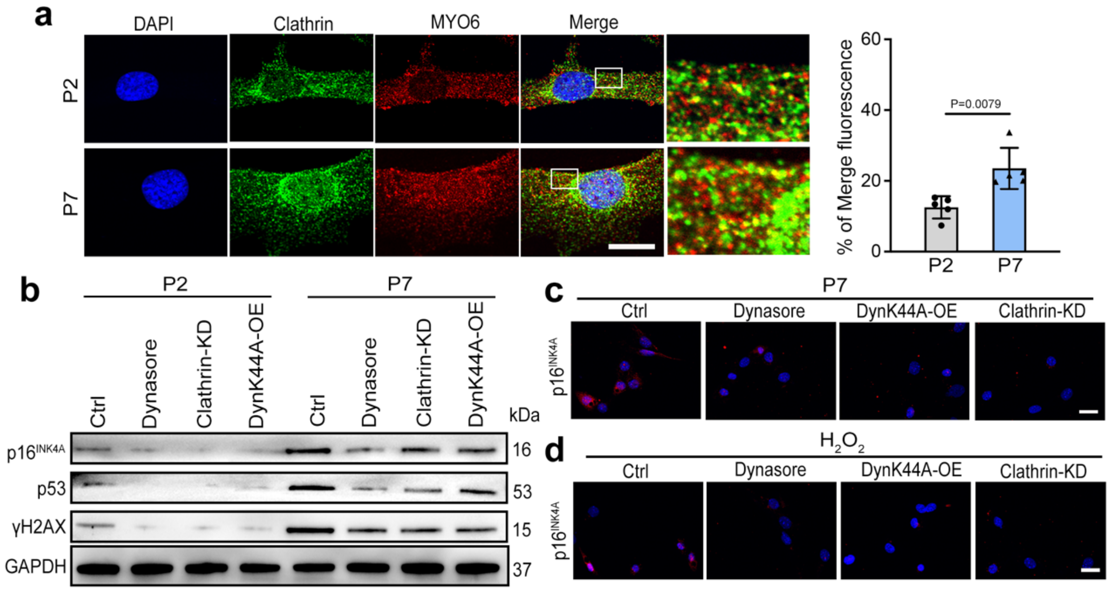

Supplementary Figure 2: CME negatively regulates cellular senescence in chondrocytes.

(a) Images and quantification of IF staining for Clathrin and MYO6 colocalization in primary chondrocytes at passage 2 or 7. n = 5, bars= 25 μm. (b) Protein levels of $p16^{INK4A}$, p53 and γH2AX in primary chondrocytes from control, Dynasore, DynK44A-OE, or Clathrin-KD at passage 2 or 7. (c) IF staining images of $p16^{INK4A}$ in primary chondrocytes from control, Dynasore, DynK44A-OE, or Clathrin-KD at passage 7. (d) IF staining images of $p16^{INK4A}$ in primary chondrocytes from control, Dynasore, DynK44A-OE, or Clathrin-KD upon H_2O_2 exposure. Data are representative of three independent experiments and are all shown as means ± SD. P values are from two-tailed Mann–Whitney U-test (a). n indicates the number of biologically independent samples or mice per group. Source data are provided as a Source Data file.

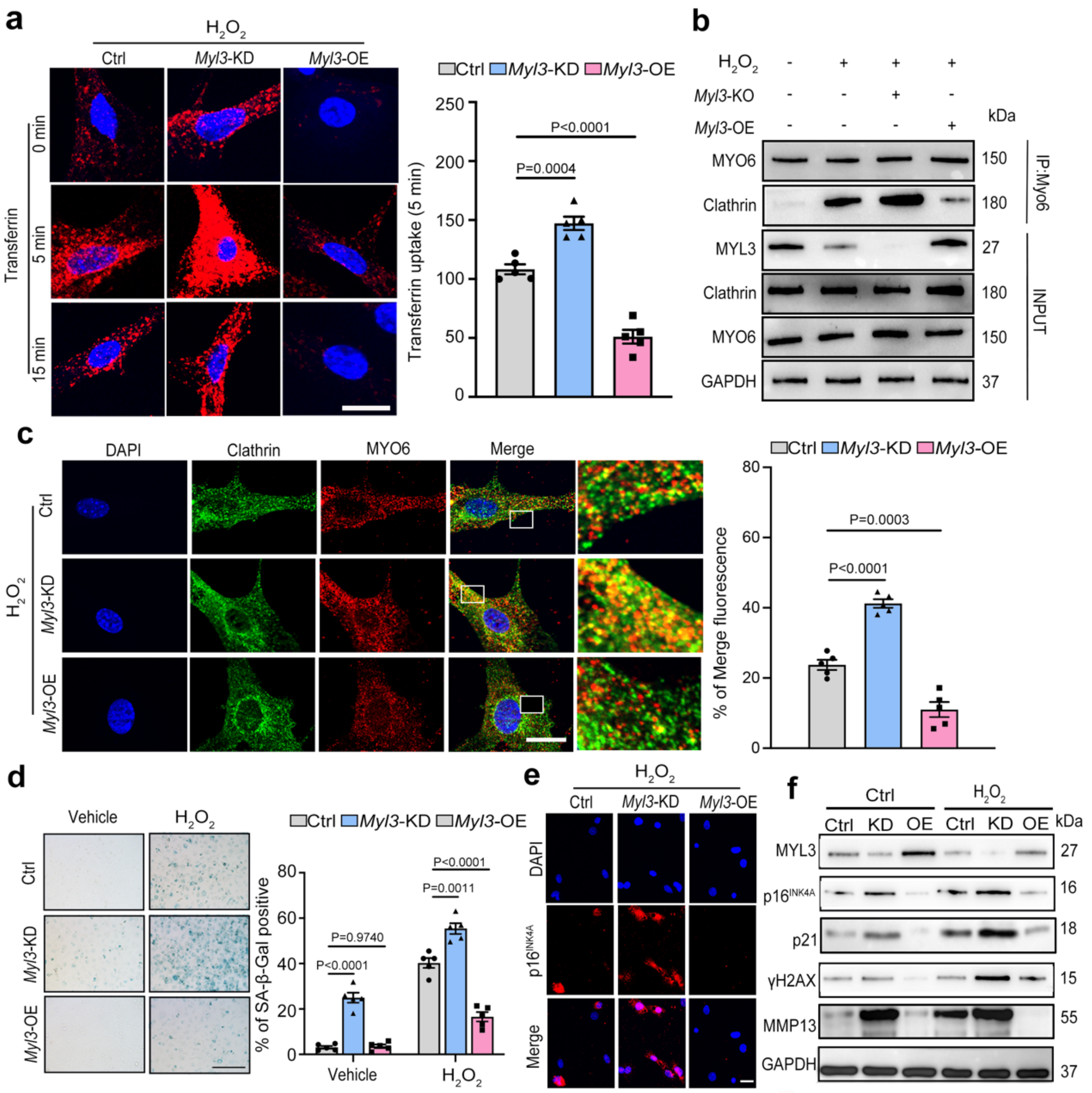

Supplementary Figure 3: MYL3 negatively regulates CME and cellular senescence in chondrocytes with H_2O_2 treatment.

(a) Images and quantification of endocytosed transferrin in mouse primary chondrocytes transfected with control, *Myl3*-KD or *Myl3*-OE and with H_2O_2 treatment. n = 5, bars= 25 μm. (b) IP detecting the binding of MYO6 and Clathrin in primary chondrocytes with vehicle or H_2O_2 treatment in conditions of control, *Myl3*-KD or *Myl3*-OE. (c) IF images and quantification of Clathrin (green) and MYO6 (red) co-localization in mouse primary chondrocytes transfected with control, *Myl3*-KD or *Myl3*-OE and with H_2O_2 treatment. n = 5, bars= 25 μm. (d) Images

and quantification of SA-β-Gal positivity in mouse primary chondrocytes transfected with control, *Myl3*-KD or *Myl3*-OE and with H_2O_2 treatment. n = 5, bars= 25 μm. (e) IF staining images of p16^{INK4A} in mouse primary chondrocytes transfected with control, *Myl3*-KD or *Myl3*-OE and with H_2O_2 treatment. n = 5, bar= 25 μm. (f) Protein levels of MYL3, p16^{INK4A}, P21, γH2AX and MMP13 in mouse primary chondrocytes transduced with control, *Myl3*-KD or *Myl3*-OE after H_2O_2 treatment. Data are representative of three independent experiments and are all shown as means ± SD. P values are from one-way ANOVA test followed by Tukey's post hoc test (a, c, d). n indicates the number of biologically independent samples or mice per group. Source data are provided as a Source Data file.

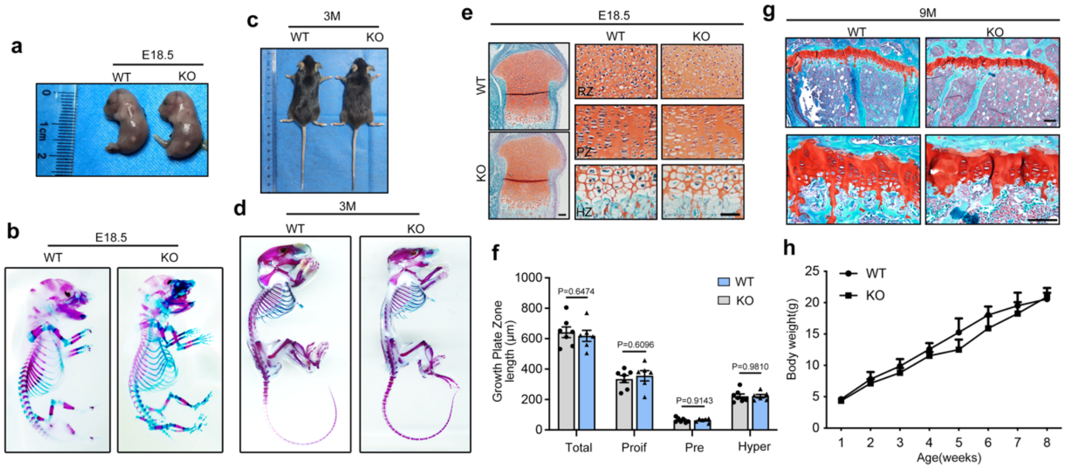

Supplementary Figure 4: Deletion of MYL3 did not affect the skeletal development of the mice.

(a) Gross appearance images of control mice and *Myl3*-KO mice at embryonic day 18.5 (E18.5). (b) Alcian blue and Alizarin red staining of skeletons of control and *Myl3*-KO mice at E18.5. (c) Gross appearance images of control and *Myl3*-KO mice at 3 months. (d) Alcian blue and Alizarin red staining of skeletons of control and *Myl3*-KO mice at 3 months. (e) Safranin-O and fast green staining of sagittal sections of tibia from control mice or *Myl3*-KO mice at E18.5. RZ, resting zone, PZ, proliferation zone, HZ, hypertrophic zone. n= 5 independent biological replicates per group, bars= 50 μm. (f) Quantification of the growth plate zone in tibiae from control mice or *Myl3*-KO mice at E18.5. (g) Safranin-O and fast green staining of sagittal sections of tibiae from control mice and *Myl3*-KO mice at 9 months. n = 5 independent biological replicates per group, bars= 50 μm. (h) Graph showing control and *Myl3*-KO mouse body weights from postnatal week 1 to week 8. n = 8 independent biological replicates per group. Data are all shown as means ± SD. P values are from two-tailed unpaired t-test. n indicates the number of biologically independent samples or mice per group. Source data are provided as a Source Data file.

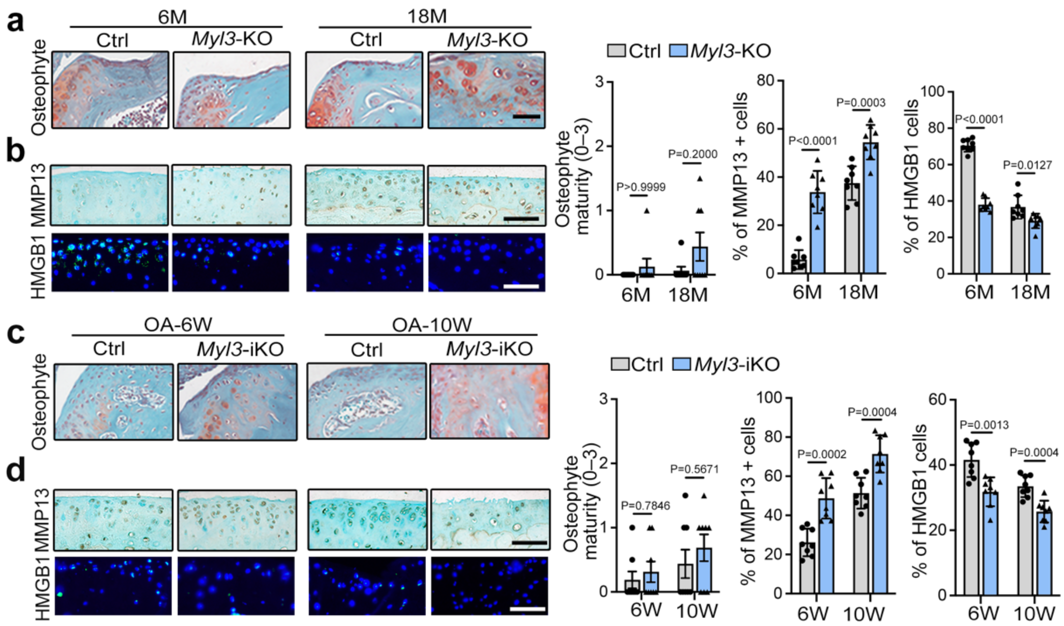

Supplementary Figure 5: Deletion of MYL3 in chondrocytes has minor effect on osteophytes formation, but increases chondrocytes catabolism.

(a) Safranin-O staining images of osteophyte and quantification of osteophyte maturity from control and *Myl3*-KO mice at 6 months and 18 months. n = 8, bars= 50 µm. (b) IHC staining of MMP13 and IF staining of HMGB1, and quantification of MMP13- and HMGB1-positive cells in articular cartilage from control and *Myl3*-KO mice at 6 months and 18 months. n = 8, bars= 50 µm. (c) Safranin-O staining images of osteophyte and quantification of osteophyte maturity from control and *Myl3*-iKO mice at 6- and 10-weeks post-surgery. n = 8, scale bars, 50 µm. (d) IHC staining of MMP13 and IF staining of HMGB1, and quantification of MMP13-, and HMGB1-positive cells in articular cartilage from control and *Myl3*-iKO mice at 6- and 10-weeks post-surgery. n = 8, bars= 50 µm. Data are all shown as means ± SD. P values are from two-tailed Mann–Whitney U-test (osteophyte maturity in a, c) and two-tailed unpaired t-test (remaining quantification). n indicates the number of biologically independent samples or mice per group. Source data are provided as a Source Data file.

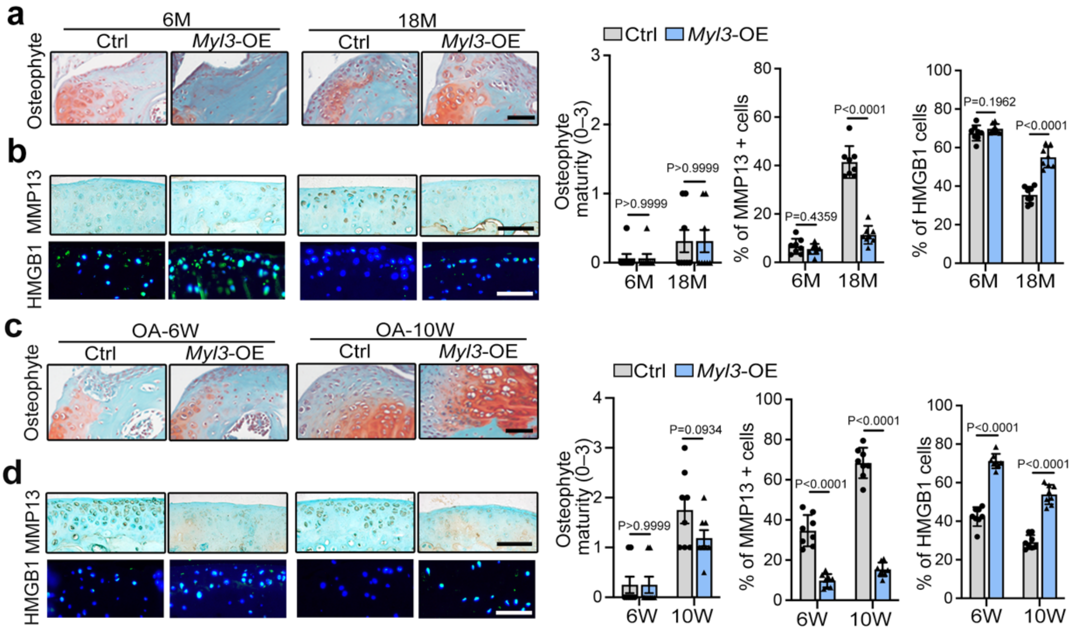

Supplementary Figure 6: Overexpression of MYL3 attenuates chondrocytes catabolism.

(a) Safranin-O staining images and quantification of osteophyte maturity from control and *Myl3*-OE mice at 6 months and 18 months. n = 8, bars= 50 μm. (b) IHC staining of MMP13 and IF staining of HMGB1, and quantification of MMP13- and HMGB1-positive cells in articular cartilage from control and *Myl3*-OE mice at 6 months and 18 months. n = 8, bars= 50 μm. (c) Safranin-O staining images and quantification of osteophyte maturity from control and *Myl3*-OE mice at 6- and 10-weeks post-surgery. n = 8, bars= 50 μm. (d) IHC staining of MMP13 and IF staining of HMGB1, and quantification of MMP13- and HMGB1-positive cells in articular cartilage from control and *Myl3*-OE mice at 6- and 10-weeks post-surgery. n = 8, bars= 50 μm. Data are all shown as means ± SD. P values are from two-tailed Mann–Whitney U-test (osteophyte maturity in a) and two-tailed unpaired t-test (remaining quantification). n indicates the number of biologically independent samples or mice per group. Source data are provided as a Source Data file.

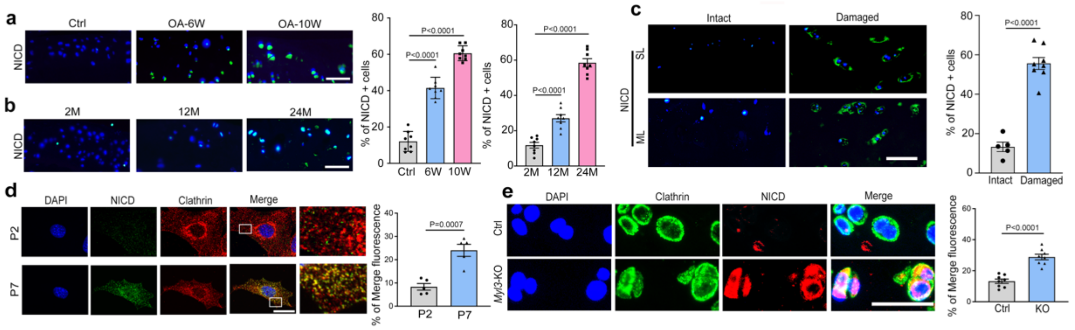

Supplementary Figure 7: Endocytosis of NICD is enhanced in senescent chondrocytes.

(a) Images and quantification of IF staining for NICD in articular cartilage from mice of control, 6- and 10- weeks post DMM surgery. n = 8, bars= 50 μm. (b) Images and quantification of IF staining for NICD in articular cartilage from 2-, 12- and 24-month-old mic. n = 8, bars= 50 μm. (c) Images and quantification of IF staining for NICD in intact (n = 5) and damaged (n = 9) articular cartilage sections collected from OA patients. Bars= 50 μm. (d) Images and quantification of IF staining for NICD (green) and Clathrin (red) colocalization in mouse primary chondrocytes from control mice at passage 2 and 7. n = 5, bars= 25 μm. (e) Images and quantification of IF staining for NICD (red) and Clathrin (green) colocalization in articular cartilage from control and *Myl3*-KO mice. n = 8, bars= 50 μm. Data are all shown as means ± SD. P values are from two-tailed unpaired t-test (c-e) and one-way ANOVA test followed by Tukey's post hoc test (a-b). n indicates the number of biologically independent samples, mice per group, or human specimens. Source data are provided as a Source Data file.

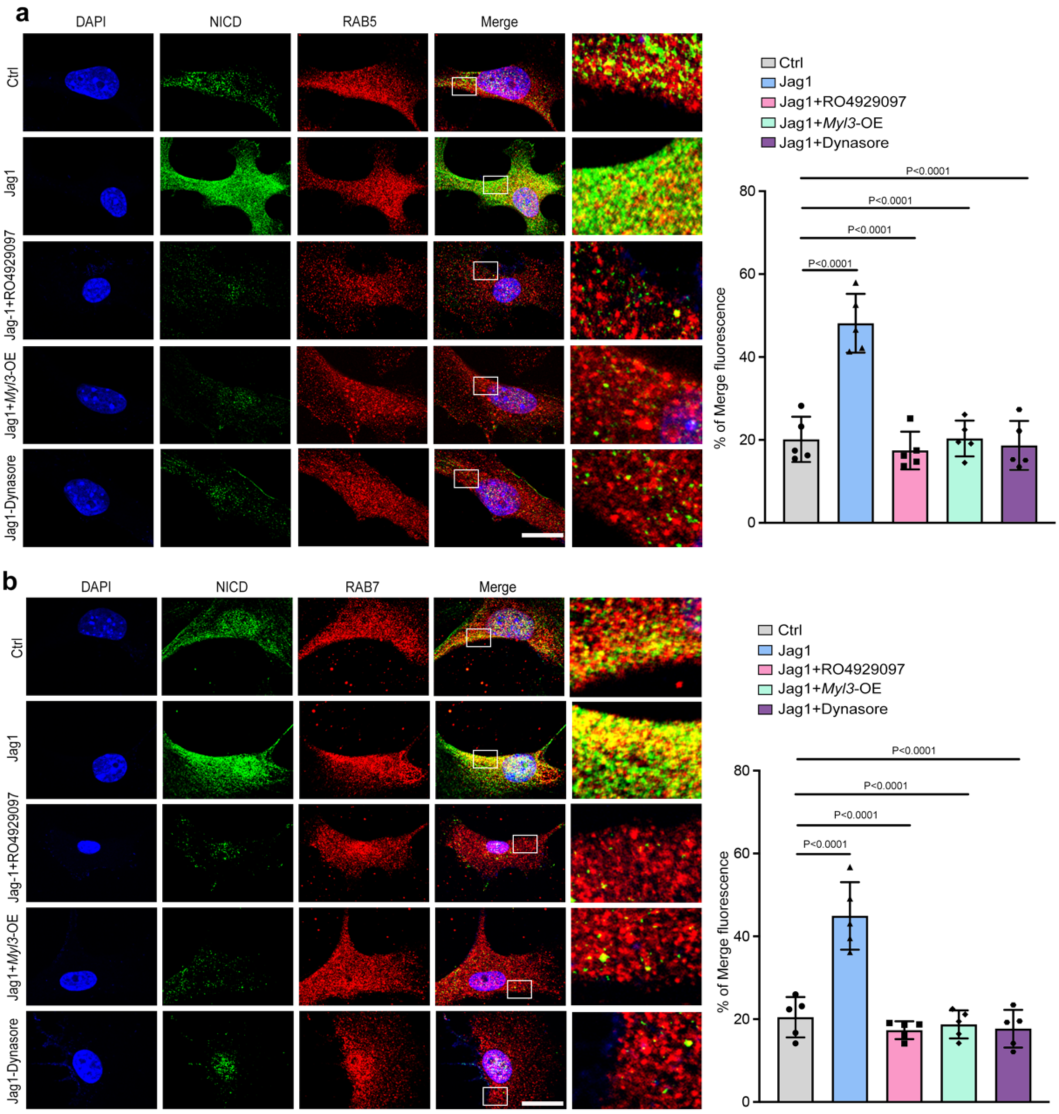

Supplementary Figure 8: Inhibition of CME downregulated co-localization of NICD and RAB5 or RAB7.

(a) Images and quantification of IF staining for NICD (green) and RAB5 (red) colocalization in mouse primary chondrocytes from control, jag1, jag1+RO4929097, jag1+*Myl3*-OE, or jag1+dynasore group. n = 5, bars= 25 µm. (b) Images and quantification of IF staining for NICD (green) and RAB7 (red) colocalization in mouse primary chondrocytes from control, jag1, jag1+RO4929097, jag1+*Myl3*-OE, or jag1+dynasore group. n = 5, bars= 25 µm. Data are all shown as means ± SD. P values are from one-way ANOVA test followed by Tukey's post hoc

test (a, b). n indicates the number of biologically independent samples or mice per group. Source data are provided as a Source Data file.

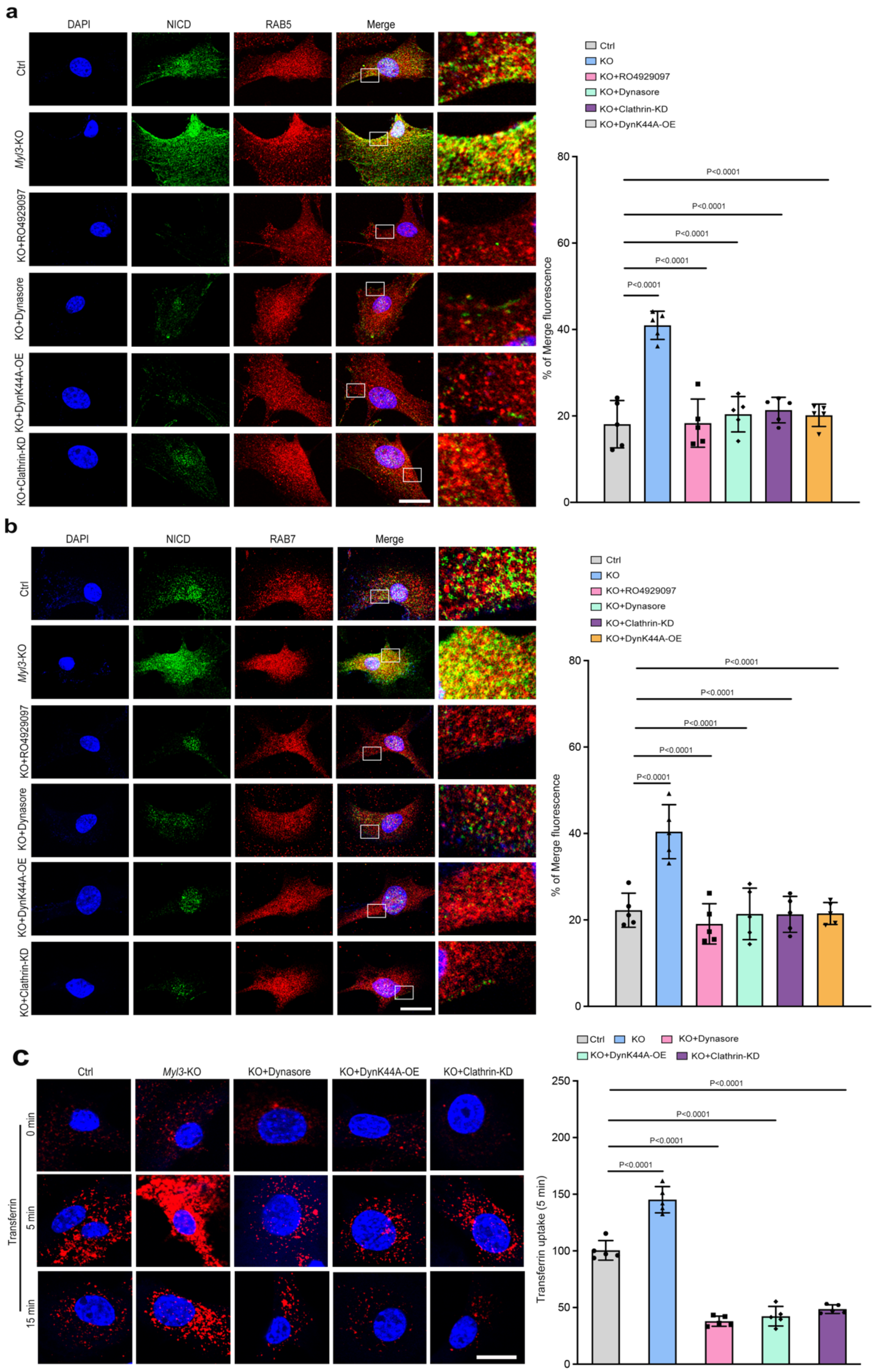

Supplementary Figure 9: Inhibition of CME prevents MYL3 loss-enhanced .Notch

signaling

(a) Images and quantification of IF staining for NICD (green) and RAB5 (red) colocalization in primary chondrocytes from control, *Myl3*-KO, KO+RO4929097, KO+Dynasore, KO+DynK44A-OE, or KO+Clathrin-KD group. n = 5, bars= 25 μm. (b) Images and quantification of IF staining for NICD (green) and RAB7 (red) colocalization in primary chondrocytes from control, *Myl3*-KO, KO+RO4929097, KO+Dynasore, KO+DynK44A-OE, or KO+Clathrin-KD group .n = 5, bars= 25 μm. (c) Images and quantification of endocytosed transferrin in mouse primary chondrocytes from control, *Myl3*-KO, KO+Dynasore, KO+DynK44A-OE, or KO+Clathrin-KD group. n = 5, bars= 25 μm. Data are all shown as means ± SD. P values are from one-way ANOVA test followed by Tukey’s post hoc test (a-c). n indicates the number of biologically independent samples or mice per group. Source data are provided as a Source Data file.

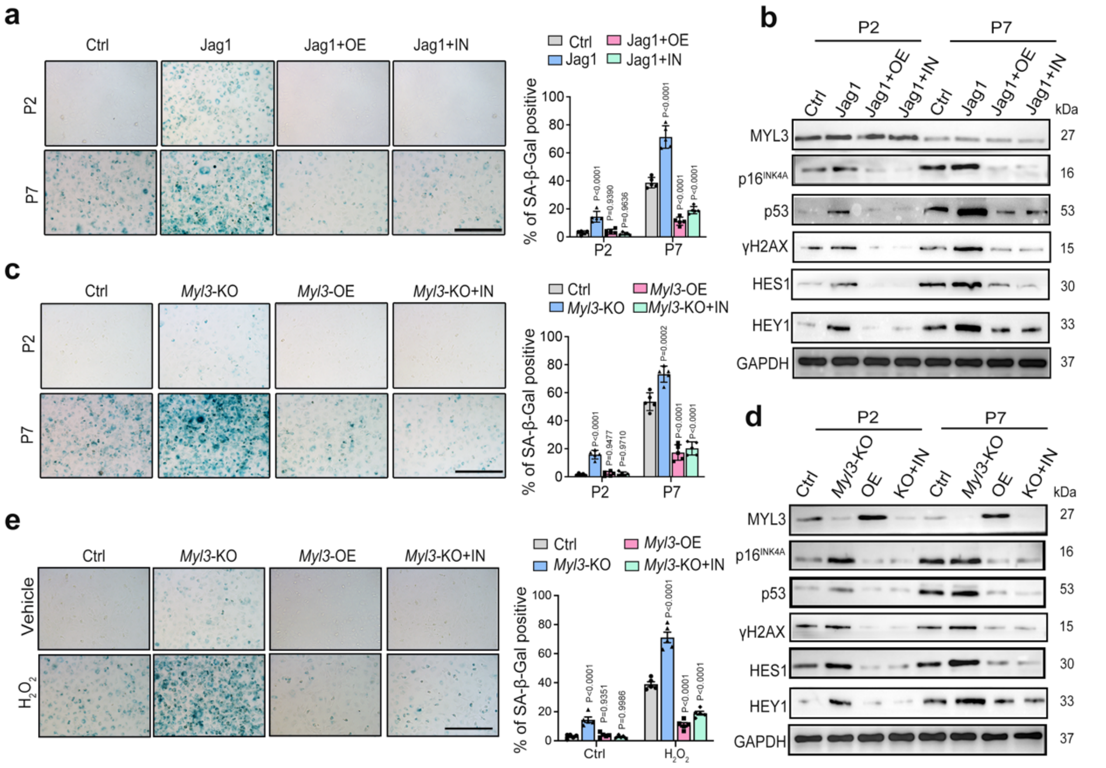

Supplementary Figure 10: Inhibition of Notch prevents MYL3 loss-enhanced chondrocyte senescence.

(a) Images and quantification of SA-β-Gal positivity, and protein levels of MYL3, $p16^{INK4A}$, p53, γH2AX, HES1, and HEY1. (b) in primary chondrocytes from control, jag1-treatment, jag1-treatment with *Myl3*-OE or RO4929097 group at passage 2 and 7. n = 5, bars= 100 μm. (c) Images and quantification of SA-β-Gal positivity, and protein levels of MYL3, $p16^{INK4A}$, p53, γH2AX, HES1 and HEY1. (d) in primary chondrocytes from control, *Myl3*-KO, *Myl3*-OE or *Myl3*-KO with RO4929097 group at passage 2 and 7. n = 5, bars= 100 μm. IN, RO4929097. (e) Images and quantification of SA-β-Gal positivity in primary chondrocytes from control, *Myl3*-KO, *Myl3*-OE or *Myl3*-KO with RO4929097 group with vehicle or H_2O_2 treatment. n = 5, bars= 100 μm. Data are representative of three independent experiments and are all shown as means ± SD. P values are from one-way ANOVA test followed by Tukey's post hoc test (a-

e). n indicates the number of biologically independent samples or mice per group. Source data are provided as a Source Data file.

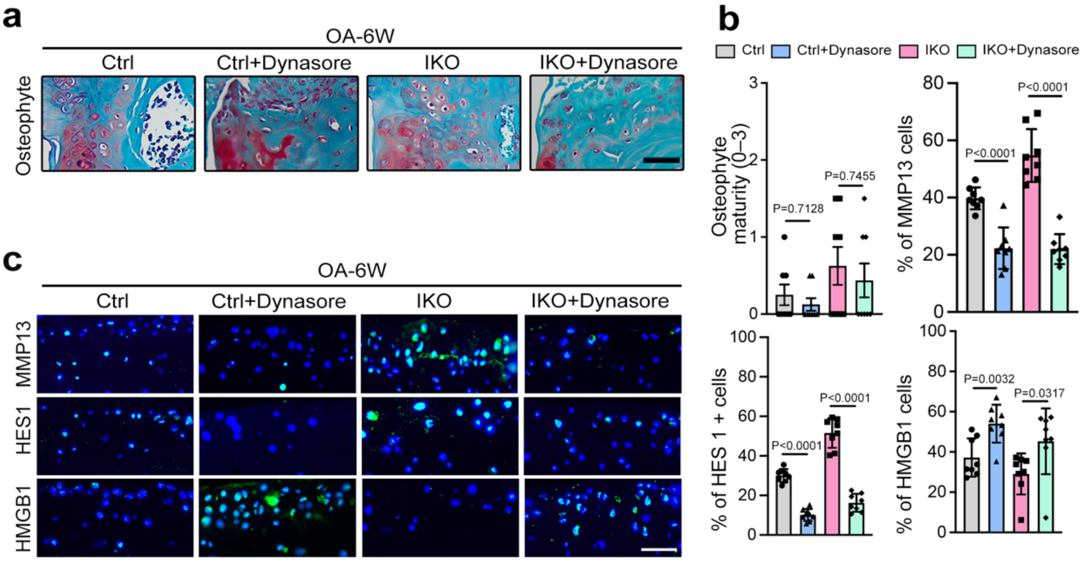

Supplementary Figure 11: Inhibition of CME prevents MYL3 loss-enhanced chondrocyte catabolism in experimental OA.

(a) Safranin-O staining images and quantification (b) of osteophyte maturity in articular cartilage from control or *Myl3*-iKO mice treated with dynasore or vehicle intra-articular injections at 6 weeks post-surgery. n = 8, bars= 50 μm. (c) IF staining and quantification (b) of MMP13, HES1 and HMGB1 in articular cartilage from control or *Myl3*-iKO mice treated with dynasore or vehicle intra-articular injections at 6 weeks post-surgery. n = 8, bars= 50 μm. Data are all shown as means ± SD. P values are from two-tailed Mann–Whitney U-test (osteophyte maturity in b) and two-tailed unpaired t-test (remaining quantification). n indicates the number of biologically independent samples or mice per group. Source data are provided as a Source Data file.

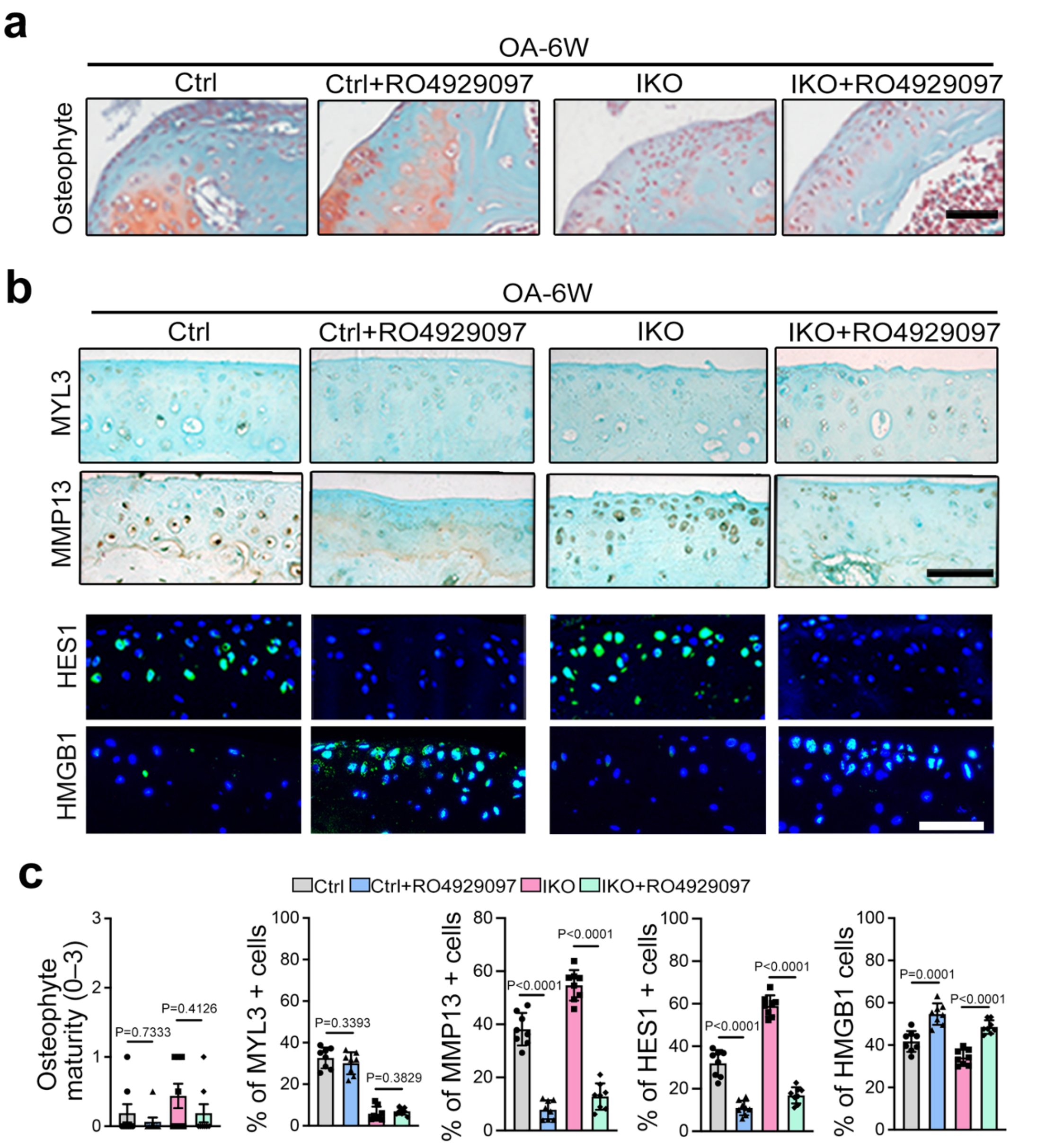

Supplementary Figure 12: Inhibition of Notch signaling prevents MYL3 loss-enhanced chondrocyte catabolism in experimental OA.

(a) Safranin-O staining images and quantification of osteophyte maturity in articular cartilage from control or *Myl3*-iKO mice treated with RO4929097 or vehicle intra-articular injections at 6 weeks post-surgery. n = 8, bars= 50 μm. (b) IHC staining and quantification (c) of MYL3 and MMP13 and IF staining of HES1 and HMGB1 in articular cartilage from control or *Myl3*-iKO mice treated with RO4929097 or vehicle intra-articular injections at 6 weeks post-surgery. n =

8, bars= 50 μm. Data are all shown as means ± SD. P values are from two-tailed Mann–Whitney U-test (osteophyte maturity in c) and two-tailed unpaired t-test (remaining quantification). n indicates the number of biologically independent samples or mice per group. Source data are provided as a Source Data file.

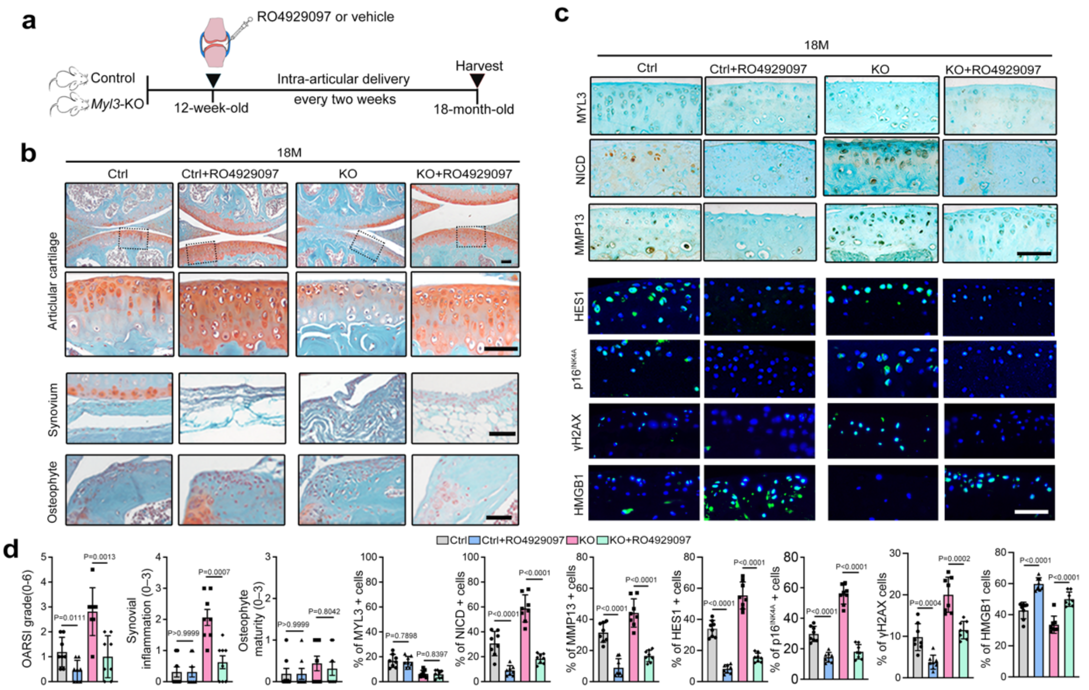

Supplementary Figure 13: Inhibition of Notch signaling prevents MYL3 loss-enhanced chondrocyte catabolism in **age-related OA.**

(a) Schematic illustration of RO4929097 or PBS delivery schedules in age-related OA model with control and *Myl3*-KO mice. (b) Safranin-O staining images and quantification of OARSI, synovial inflammation and osteophyte maturity scores, (c)images and quantification of IHC staining for MYL3, NICD and MMP13 and IF staining for HES1, $p16^{INK4A}$ γH2AX and HMGB1 (d) in sagittal sections of joints from control or *Myl3*-KO mice treated with RO4929097 or vehicle intra-articular injections at 18 months. n = 8, bars= 50 µm. Data are all shown as means ± SD. P values are from two-tailed Mann–Whitney U-test (synovial inflammation scores, osteophyte maturity in d) and two-tailed unpaired t-test (remaining quantification). n indicates the number of biologically independent samples or mice per group. Source data are provided as a Source Data file.

Supplementary Table 1 Top-regulated proteins in mouse cartilage (2M vs 12M)

Protein	L12M/L2M Ratio	P Value
**Down-regulated**		
Chondroadherin-like protein	0.072566144	6.77046E-06
Myosin light chain 3	0.104858036	6.37052E-06
Matrilin-3	0.134711892	3.19172E-06
EGF-containing fibulin-like extracellular matrix protein 1	0.143689296	3.39477E-06
Collagen alpha-1(IX) chain	0.188178383	1.19527E-06
Serpin H1 OS=Mus musculus	0.193523181	1.99203E-06
Peptidyl-prolyl cis-trans isomerase FKBP10	0.19700577	1.98464E-05
Collagen alpha-2(IX) chain	0.197913587	2.84284E-07
Peptidyl-prolyl cis-trans isomerase C	0.210569689	1.78986E-06
Prolyl 4-hydroxylase subunit alpha-1	0.216737455	0.000239224
**Up-regulated**		
Ig gamma-1 chain C region secreted form	7.406613912	0.000290942
Immunoglobulin heavy constant gamma 2B	6.416236099	2.06575E-05
Ig gamma-2A chain C region secreted form	5.878273063	1.26822E-05
Leucine-rich repeat flightless-interacting protein 1	4.712600886	0.002773091
Fatty acid-binding protein 5	3.98908218	3.38281E-06
Immunoglobulin heavy constant mu	3.075224876	3.48689E-06
Chitinase-like protein 3 OS=Mus musculus	2.990573995	2.90576E-07
Peptidoglycan recognition protein 1	2.918847987	0.000866945
Dedicator of cytokinesis protein 8	2.804471993	0.011982775
Serine/threonine-protein kinase 10	2.671812287	0.015861807

Supplementary Table 2 Top 20 up-regulated genes in mouse cartilage (Ctrl vs KO)		
**Gene**	**Log2(FC)**	**P Value**
**Up-regulated**		
Gm14440	10.86109	4.97E-28
Zfp993	9.204571	0.000979
Cd72	8.451211	0.000245
Pla2g4b	8.228819	0.00049
Ccrl2	7.965784	0.007823
Hvcn1	7.78136	0.003913
Tmem200a	7.714246	0.000979
Pcdha4	7.569856	6.14E-05
Gm6502	4.285402	0.003913
Adgrd1	2.807355	1.02E-14
Gm2042	2.676461	0.000919
Col4a2	2.643856	0.000182
Cfh	2.608809	8.12E-08
Ptgdr2	2.584963	0.012969
Duox1	2.5025	0.007406
Colca2	2.428843	0.007406
Cdh15	2.382003	5.17E-45
Slc3a1	2.268489	0.004364
Hes1	2.263776	5.27E-12
Etv5	2.146841	5.42E-06

Supplementary Table 3 Top 20 of Pathway Enrichment (Ctrl vs KO)

Pathway	Q value	P Value
ECM-receptor interaction	0.149397	0.00344948
Protein digestion and absorption	0.149397	0.00462909
Thiamine metabolism	0.149397	0.00565968
Notch signaling pathway	0.149397	0.00574604
Regulation of lipolysis in adipocyte	0.174593	0.00839388
Long-term depression	0.184753	0.01065884
Platelet activation	0.20555	0.01383512
Focal adhesion	0.219273	0.01686717
Salivary secretion	0.240408	0.02080454
Tyrosine metabolism	0.323053	0.03387938
Circadian entrainment	0.323053	0.03770245
Intestinal immune network for IgA production	0.323053	0.03894907
PI3K-Akt signaling pathway	0.323053	0.04266408
Axon guidance	0.323053	0.0434879
Thyroid hormone signaling pathway	0.398025	0.0574074
Vitamin B6 metabolism	0.434203	0.06680038
Regulation of actin cytoskeleton	0.484763	0.07924008
Apelin signaling pathway	0.501746	0.08684067
Drug metabolism - cytochrome P450	0.507491	0.09271477
Glycosaminoglycan biosynthesis - keratan sulfate	0.530385	0.1019972

Uncropped images of the blots

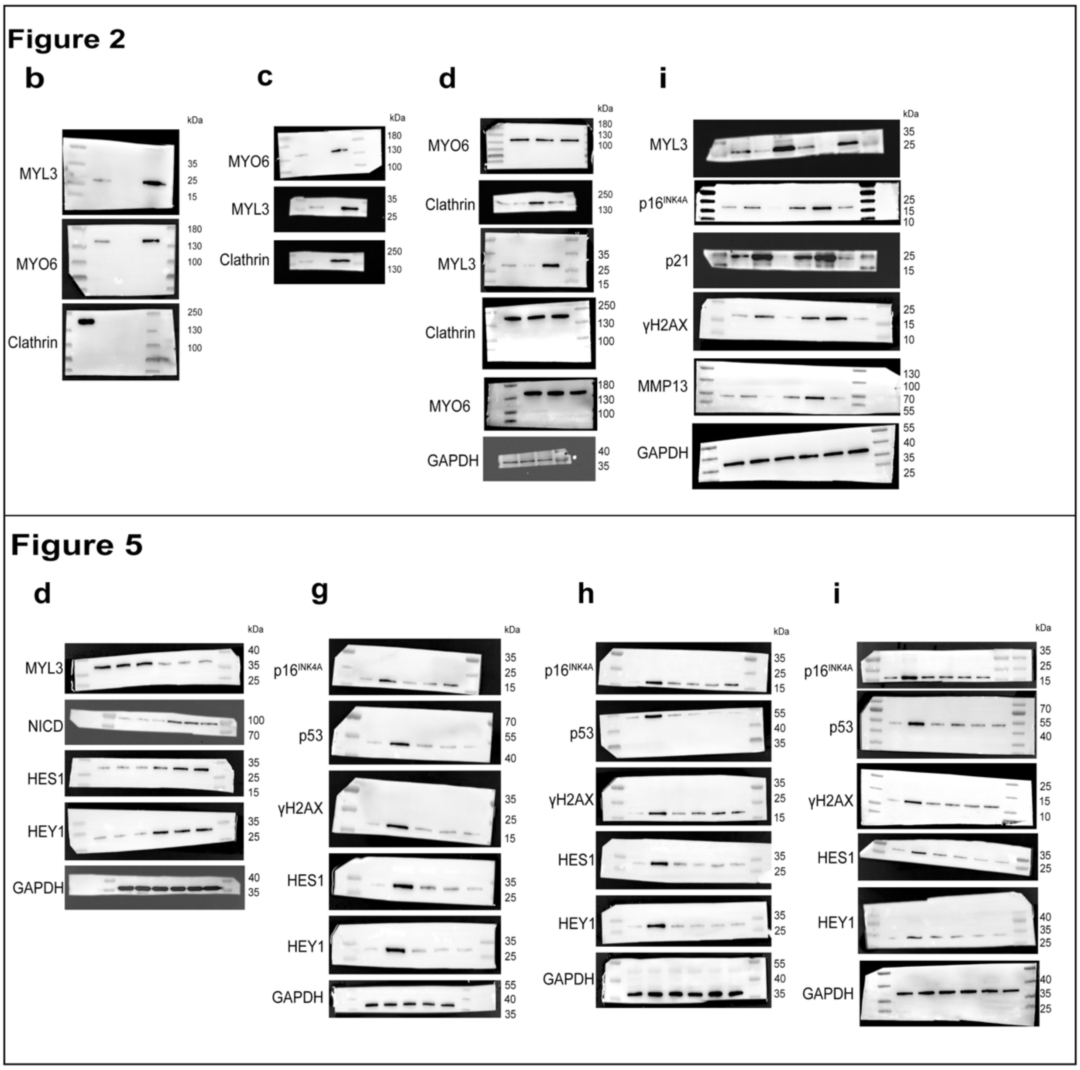

Supplementary Figure 1

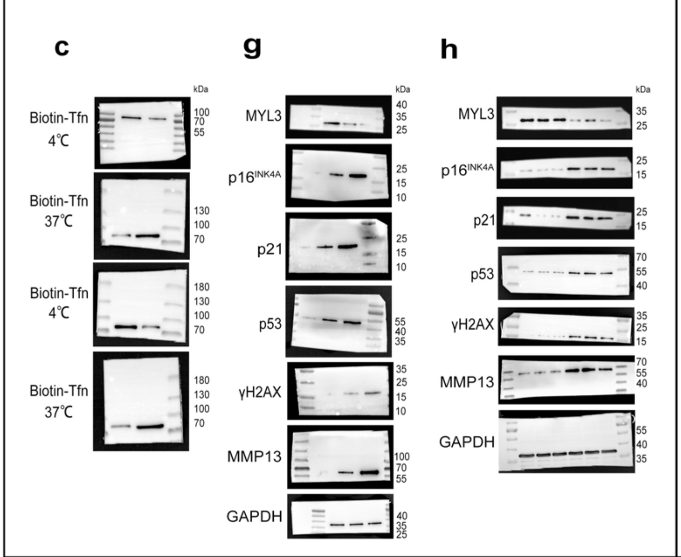

Supplementary Figure 2

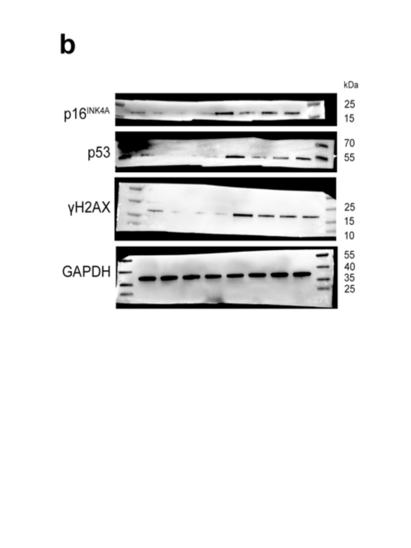

Supplementary Figure 3

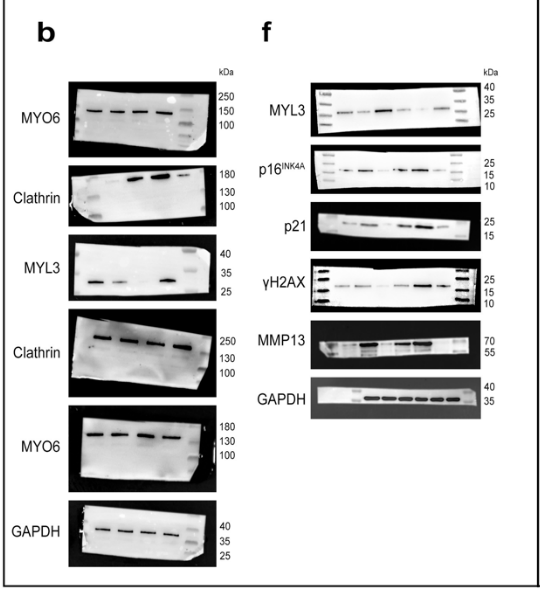

Supplementary Figure 10

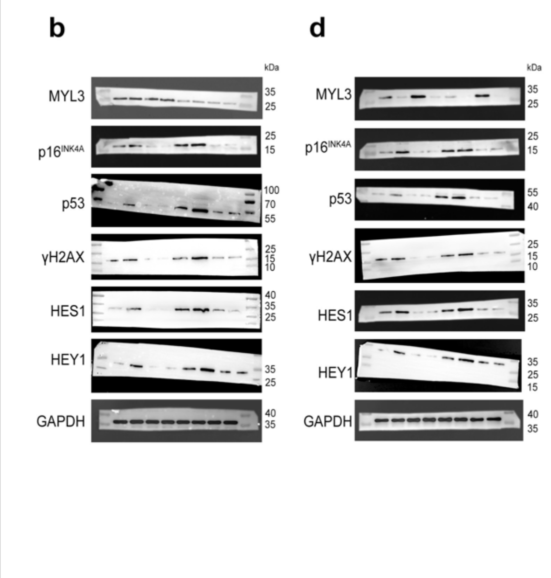

Supplement: Supplementary file 1 — Supplementary Information [file 41467_2023_41858_MOESM1_ESM.pdf]
