# Supplementary material for: MYL3 protects chondrocytes from senescence by inhibiting clathrin-mediated endocytosis and activating of Notch signaling
Source: Nat Commun. 2023 Oct 4;14:6190. doi: 10.1038/s41467-023-41858-7 (PMC10550997; doi:10.1038/s41467-023-41858-7)
Supplement: Supplementary file 3 — Reporting Summary [file 41467_2023_41858_MOESM3_ESM.pdf]

nature portfolio

Corresponding author(s): Kai Li; Xiaochun Bai

Last updated by author(s): Sep 8, 2023

Reporting Summary

Nature Portfolio wishes to improve the reproducibility of the work that we publish. This form provides structure for consistency and transparency in reporting. For further information on Nature Portfolio policies, see our Editorial Policies and the Editorial Policy Checklist.

Statistics

For all statistical analyses, confirm that the following items are present in the figure legend, table legend, main text, or Methods section.

n/a	Confirmed	
☐	☒	The exact sample size (*n*) for each experimental group/condition, given as a discrete number and unit of measurement
☐	☒	A statement on whether measurements were taken from distinct samples or whether the same sample was measured repeatedly
☐	☒	The statistical test(s) used AND whether they are one- or two-sided<br>*Only common tests should be described solely by name; describe more complex techniques in the Methods section.*
☒	☐	A description of all covariates tested
☒	☐	A description of any assumptions or corrections, such as tests of normality and adjustment for multiple comparisons
☐	☒	A full description of the statistical parameters including central tendency (e.g. means) or other basic estimates (e.g. regression coefficient) AND variation (e.g. standard deviation) or associated estimates of uncertainty (e.g. confidence intervals)
☐	☒	For null hypothesis testing, the test statistic (e.g. *F*, *t*, *r*) with confidence intervals, effect sizes, degrees of freedom and *P* value noted<br>*Give P values as exact values whenever suitable.*
☒	☐	For Bayesian analysis, information on the choice of priors and Markov chain Monte Carlo settings
☒	☐	For hierarchical and complex designs, identification of the appropriate level for tests and full reporting of outcomes
☒	☐	Estimates of effect sizes (e.g. Cohen's *d*, Pearson's *r*), indicating how they were calculated

Our web collection on statistics for biologists contains articles on many of the points above.

Software and code

Policy information about availability of computer code

Data collection	LightCycler® 96 software 1.1 used for quantitative polymerase chain reaction(QPCR), CellSens (v4.1) software used for sections imaging, FV10-ASW Viewer (v4.2) software used for sections and cells imaging, Tanon image (v1.0) software and geneSys (v1.8.5) used for imaging and data acquisition for western blot.
Data analysis	All data are presented as means ± SD using SPSS version 20.0 software, and graphs were generated using GraphPad Prism 8.0. Image Pro Plus (v6.0) software and Image J (v1.8) used for image analysis. RT-PCR data was analyzed in Microsoft Excel version 16.36. GO enrichment analysis was implemented using the GOseq R Package and DAVID online tool (https://david.ncifcrf.gov/). Pathways of differentially expressed genes were analyzed using the KEGG database (http://www.kegg.jp/kegg/) and GESA software (v4.0.3).The separated peptides underwent analysis by an Orbitrap Exploris 480 equipped with a nano-electrospray ion source. The MaxQuant search engine (v.1.6.15.0) was utilized to process the MS/MS data and generate the desired results.

For manuscripts utilizing custom algorithms or software that are central to the research but not yet described in published literature, software must be made available to editors and reviewers. We strongly encourage code deposition in a community repository (e.g. GitHub). See the Nature Portfolio guidelines for submitting code & software for further information.

Data

Policy information about availability of data

All manuscripts must include a data availability statement. This statement should provide the following information, where applicable:

- Accession codes, unique identifiers, or web links for publicly available datasets
- A description of any restrictions on data availability
- For clinical datasets or third party data, please ensure that the statement adheres to our policy

The original RNA-seq data generated in this study have been deposited in the GEO database under accession code GSE232325. The mass spectrometry proteomics data have been deposited to the ProteomeXchange Consortium via the PRIDE partner repository with the dataset identifier PXD044684. All other relevant data supporting the findings of this study are available within the article and its Supplementary Information file. Source data are provided with this paper.

Research involving human participants, their data, or biological material

Policy information about studies with human participants or human data. See also policy information about sex, gender (identity/presentation), and sexual orientation and race, ethnicity and racism.

Reporting on sex and gender	Human OA cartilage was obtained from patients undergoing total knee replacement surgery in the Department of Orthopedic Surgery at the Third Affiliated Hospital of Southern Medical University (Guangzhou, China) (n = 8; five males and three females). Control cartilage was collected from traffic incident victims with no history of arthritic disease (n = 5; three males and two females).
Reporting on race, ethnicity, or other socially relevant groupings	Human OA cartilage was obtained from patients undergoing total knee replacement surgery in the Department of Orthopedic Surgery at the Third Affiliated Hospital of Southern Medical University (Guangzhou, China) (n = 8; aged 67 ± 2.03 years; five males and three females). OA was confirmed in X-ray images of the knee joint or in full-length X-ray images of both lower limbs. Control cartilage was collected from traffic incident victims with no history of arthritic disease (n = 5; aged 54 ± 8.48 years; three males and two females). Race or ethnicity was not used as a proxy for socioeconomic status. There was no self-selection bias in this study and each patient provided informed consent before human tissue samples were harvested.
Population characteristics	Human OA cartilage was obtained from patients undergoing total knee replacement surgery in the Department of Orthopedic Surgery at the Third Affiliated Hospital of Southern Medical University (Guangzhou, China) (n = 8; aged 67 ± 2.03 years). Control cartilage was collected from traffic incident victims with no history of arthritic disease (n = 5; aged 54 ± 8.48 years).
Recruitment	All patients were recruited at the Department of Orthopedic Surgery at the Third Affiliated Hospital of Southern Medical University (Guangzhou, China). Patients (n = 8; aged 67 ± 2.03 years; five males and three females) undergoing total knee replacement surgery were recruited. OA was confirmed in X-ray images of the knee joint or in full-length X-ray images of both lower limbs. The Patients (n = 5; aged 54 ± 8.48 years; three males and two females) with traffic incident victims with no history of arthritic disease were recruited to provide control cartilage. There was no self-selection bias in this study. Written informed consent was obtained from all subjects before human tissue samples were harvested.
Ethics oversight	All studies were approved by the Medical Ethics Committee of The Third Affiliated Hospital of Southern Medical University. And each patient provided informed consent before human tissue samples were harvested.

Note that full information on the approval of the study protocol must also be provided in the manuscript.

Field-specific reporting

Please select the one below that is the best fit for your research. If you are not sure, read the appropriate sections before making your selection.

☒ Life sciences ☐ Behavioural & social sciences ☐ Ecological, evolutionary & environmental sciences

For a reference copy of the document with all sections, see nature.com/documents/nr-reporting-summary-flat.pdf

Life sciences study design

All studies must disclose on these points even when the disclosure is negative.

Sample size	Sample size for each experiment is indicated in the legend. Prior to commencing the study, no calculations for determining sample size were performed. Sample sizes were determined based on previous studies: PMID 36496445, 35140209, 32647171. Sample sizes were chosen to ensure an adequate statistical power based on size of effects observed and reaching statistical significance based on the available samples. All experiments were conducted on at least three independent biological replicates, including all histology and immunohistochemistry experiments.
Data exclusions	No data was excluded.
Replication	All experiments were performed in at least triplicate and reproducible across independent experiments. Western blot pictures are from a representative experiment and the number of independent repeats is clearly indicated in the figure legends. Number of biological replicates

	are indicated in figures.
Randomization	For in vitro experiments, cell cultures are randomly assigned to each experimental group. Wild type and transgenic mice were randomly assigned into each experimental group with various treatments. Randomization was performed when performing DMM surgeries on mice, with the surgeon unaware of the genotype of the mice. Littermate mice were ultimately compared based on genotype. All allocations were randomized in this study.
Blinding	For the mice surgeries and intraarticular injections, the operators were blinded to the group information. The investigators used sample ID and were not given grouping information during data collection. For histological analysis, the investigation were blinded to the group information, including genotype, treatment or surgical condition of mice. WB,RT-PCR, IHC and IF were performed by participants other than the experiment designer.

Reporting for specific materials, systems and methods

We require information from authors about some types of materials, experimental systems and methods used in many studies. Here, indicate whether each material, system or method listed is relevant to your study. If you are not sure if a list item applies to your research, read the appropriate section before selecting a response.

Materials & experimental systems

n/a	Involved in the study
☐	☒ Antibodies
☒	☐ Eukaryotic cell lines
☒	☐ Palaeontology and archaeology
☐	☒ Animals and other organisms
☒	☐ Clinical data
☒	☐ Dual use research of concern
☒	☐ Plants

Methods

n/a	Involved in the study
☒	☐ ChIP-seq
☒	☐ Flow cytometry
☒	☐ MRI-based neuroimaging

Antibodies

Antibodies used

IHC or IF
peroxidase-conjugated affinipure goat anti-mouse IgG (1:500, 115-035-003, Jackson ImmunoResearch, USA), peroxidase-conjugated affiniPure goat anti-rabbit IgG (1:500, 111-035-003, Jackson ImmunoResearch), anti-rabbit Alexa Fluor™ 488 (1:400, a11008, Invitrogen, USA), anti-rabbit Alexa Fluor™ 594 (1:400, a11012, Invitrogen), anti-mouse Alexa Fluor™ 488 (1:400, a21202, Invitrogen), anti-mouse Alexa Fluor™ 594 (1:400, a21203, Invitrogen) , mouse monoclonal anti-MYL3 (1:200, MLM527, ab680; Abcam, Cambridge, UK), rabbit polyclonal anti-MYL3 (1:100, 10913-1-AP, Proteintech, Rosemont, IL, USA) , mouse monoclonal anti-p16 (1:200, 2D9A12, ab54210, Abcam) , rabbit polyclonal anti-p16 (1:200, A0262, Abclonal, Wuhan, CN) , rabbit monoclonal anti-γH2AX (1:200, EP8542Y, ab81299, Abcam) , rabbit polyclonal anti-HMGB1 (1:200, 10829-1-A, Proteintech) , rabbit polyclonal anti-MMP13 (1:200, A1606, Abclonal) , rabbit polyclonal anti-NICD (1:200, ab8925, Abcam) , rabbit polyclonal anti-MYO6(1:200, 26778-1-AP, Proteintech) , rabbit polyclonal anti-MYO6(1:200, ab230478, Abcam), rabbit polyclonal anti-clathrin (1:200, ab21679, Abcam), mouse monoclonal anti-clathrin (1:200, X22, ab2731, Abcam), mouse monoclonal anti-RAB5 (1:200, 3A4, ab66746, Abcam) , mouse monoclonal anti-RAB7 (1:200, Rab7-117, ab50533, Abcam), rabbit monoclonal anti-HES1 (1:100, ARC0513, A0925, Abclonal)

Western blotting
peroxidase-conjugated affinipure goat anti-mouse IgG (1:5000, 115-035-003, Jackson ImmunoResearch, USA), peroxidase-conjugated affinipure goat anti-rabbit IgG (1:5000, 111-035-003, Jackson ImmunoResearch), rabbit polyclonal anti-MYL3 (1:1000, 10913-1-AP, Proteintech), rabbit polyclonal anti-p16 (1:1000, A0262, Abclonal), rabbit polyclonal anti-MMP13 (1:2000, A1606, Abclonal), rabbit polyclonal anti-p21 (1:2000, A1483, Abclonal), mouse monoclonal anti-p53 (1:2000, Clone 1C12, 2524S, Cell Signaling Technology, Danvers, USA), rabbit monoclonal anti-γH2AX (1:1000, EP8542Y, ab81299, Abcam), rabbit polyclonal anti-MYO6 (1:1000, 26778-1-AP, Proteintech), rabbit polyclonal anti-MYO6 (1:1000, ab230478, Abcam), rabbit polyclonal anti-Clathrin (1:1000, ab21679, Abcam), rabbit polyclonal anti-HEY1 (1:1000, A16110, Proteintech) and rabbit monoclonal anti-HES1 (1:1000, A0925, Abclonal).

Validation

IHC or IF
peroxidase-conjugated affinipure goat anti-mouse IgG (1:500, 115-035-003, Jackson ImmunoResearch, USA)
https://www.jacksonimmuno.com/catalog/products/115-035-003
peroxidase-conjugated affiniPure goat anti-rabbit IgG (1:500, 111-035-003, Jackson ImmunoResearch)
https://www.jacksonimmuno.com/catalog/products/111-035-003
anti-rabbit Alexa Fluor™ 488 (1:400, a11008, Invitrogen, USA)
https://www.thermofisher.cn/cn/zh/antibody/product/Goat-anti-Rabbit-IgG-H-L-Cross-Adsorbed-Secondary-Antibody-Polyclonal/A-11008
anti-rabbit Alexa Fluor™ 594 (1:400, a11012, Invitrogen)
https://www.thermofisher.cn/cn/zh/antibody/product/Goat-anti-Rabbit-IgG-H-L-Cross-Adsorbed-Secondary-Antibody-Polyclonal/A-11012
anti-mouse Alexa Fluor™ 488 (1:400, a21202, Invitrogen)
https://www.thermofisher.cn/cn/zh/antibody/product/Donkey-anti-Mouse-IgG-H-L-Highly-Cross-Adsorbed-Secondary-Antibody-Polyclonal/A-21202
anti-mouse Alexa Fluor™ 594 (1:400, a21203, Invitrogen)
https://www.thermofisher.cn/cn/zh/antibody/product/Donkey-anti-Mouse-IgG-H-L-Highly-Cross-Adsorbed-Secondary-Antibody-

Polyclonal/A-21203
mouse monoclonal anti-MYL3 (1:200, MLM527, ab680; Abcam, Cambridge, UK)
https://www.abcam.cn/products/primary-antibodies/myosin-light-chain-3-antibody-mlm527-ab680.html
rabbit polyclonal anti-MYL3 (1:100, 10913-1-AP, Proteintech, Rosemont, IL, USA)
https://www.ptgcn.com/products/MYL3-Antibody-10913-1-AP.htm
mouse monoclonal anti-p16 (1:200, 2D9A12, ab54210, Abcam)
https://www.abcam.cn/products/primary-antibodies/cdkn2ap16ink4a-antibody-2d9a12-ab54210.html
rabbit polyclonal anti-p16 (1:200, A0262, Abclonal, Wuhan, CN)
https://abclonal.com.cn/catalog/A0262
rabbit monoclonal anti-γH2AX (1:200, EP8542Y, ab81299, Abcam)
https://www.abcam.cn/products/primary-antibodies/gamma-h2ax-phospho-s139-antibody-ep8542y-ab81299.html
rabbit polyclonal anti-HMGB1 (1:200, 10829-1-A, Proteintech)
https://www.ptgcn.com/products/HMGB1-Antibody-10829-1-AP.htm
rabbit polyclonal anti-MMP13 (1:200, A1606, Abclonal)
https://abclonal.com.cn/catalog/A1606
rabbit polyclonal anti-NICD (1:200, ab8925, Abcam)
https://www.abcam.cn/products/primary-antibodies/activated-notch1-antibody-ab8925.html
rabbit polyclonal anti-MYO6(1:200, 26778-1-AP, Proteintech)
https://www.ptgcn.com/products/MYO6-Antibody-26778-1-AP.htm
rabbit polyclonal anti-MYO6(1:200, ab230478, Abcam)
https://www.abcam.cn/products/primary-antibodies/myo6-antibody-ab230478.html
rabbit polyclonal anti-clathrin (1:200, ab21679, Abcam)
https://www.abcam.cn/products/primary-antibodies/clathrin-heavy-chain-antibody-ab21679.html
mouse monoclonal anti-clathrin (1:200, X22, ab2731, Abcam)
https://www.abcam.cn/products/primary-antibodies/clathrin-heavy-chain-antibody-x22-ab2731.html
mouse monoclonal anti-RAB5 (1:200, 3A4, ab66746, Abcam)
https://www.abcam.cn/products/primary-antibodies/rab5a-antibody-3a4-early-endosome-marker-ab66746.html
mouse monoclonal anti-RAB7 (1:200, Rab7-117, ab50533, Abcam)
https://www.abcam.cn/products/primary-antibodies/rab7-antibody-rab7-117-late-endosome-marker-ab50533.html
rabbit monoclonal anti-HES1 (1:100, ARC0513, A0925, Abclonal)
https://abclonal.com.cn/catalog/A0925

WB
peroxidase-conjugated affinipure goat anti-mouse IgG (1:5000, 115-035-003, Jackson ImmunoResearch, USA)
https://www.jacksonimmuno.com/catalog/products/115-035-003
peroxidase-conjugated affiniPure goat anti-rabbit IgG (1:5000, 111-035-003, Jackson ImmunoResearch)
rabbit polyclonal anti-MYL3 (1:1000, 10913-1-AP, Proteintech)
https://www.ptgcn.com/products/MYL3-Antibody-10913-1-AP.htm
rabbit polyclonal anti-p16 (1:1000, A0262, Abclonal)
https://abclonal.com.cn/catalog/A0262
rabbit polyclonal anti-MMP13 (1:2000, A1606, Abclonal)
https://abclonal.com.cn/catalog/A1606
rabbit polyclonal anti-p21 (1:2000, A1483, Abclonal)
https://abclonal.com.cn/catalog/A1483
mouse monoclonal anti-p53 (1:2000, Clone 1C12, 2524S, Cell Signaling Technology, Danvers, USA)
https://www.cellsignal.cn/products/primary-antibodies/p53-1c12-mouse-mab/2524?site-search-type=Products&N=4294956287&Ntt=2524s&fromPage=plp&_requestid=47930
rabbit monoclonal anti-γH2AX (1:1000, EP8542Y, ab81299, Abcam)
https://www.abcam.cn/products/primary-antibodies/gamma-h2ax-phospho-s139-antibody-ep8542y-ab81299.html
rabbit polyclonal anti-MYO6(1:1000, 26778-1-AP, Proteintech)
https://www.ptgcn.com/products/MYO6-Antibody-26778-1-AP.htm
rabbit polyclonal anti-MYO6(1:1000, ab230478, Abcam)
https://www.abcam.cn/products/primary-antibodies/myo6-antibody-ab230478.html
rabbit polyclonal anti-Clathrin (1:1000, ab21679, Abcam)
https://www.abcam.cn/products/primary-antibodies/clathrin-heavy-chain-antibody-ab21679.html
rabbit polyclonal anti-HEY1 (1:1000, A16110, Proteintech)
https://abclonal.com.cn/catalog/A16110
rabbit monoclonal anti-HES1 (1:1000, A0925, Abclonal)
https://abclonal.com.cn/catalog/A0925

Animals and other research organisms

Policy information about studies involving animals; ARRIVE guidelines recommended for reporting animal research, and Sex and Gender in Research

Laboratory animals

All animal experiments were approved by the Animal Care and Use Committee of Southern Medical University (Guangzhou, China). Twelve-week-old male C57BL/6J mice used for the experimental OA and 6-, 15- and 18-month-old male C57BL/6J mice used for aged-OA studies were purchased from the Laboratory Animal Center of Southern Medical University. For the Myl3-flox mice, embryonic stem cell clone (Clone NO. HEPD0622_5_G02) was purchased from Cambridge-Suda (Cam-SU) Genomic Resource Center (Suzhou, China). Germline-transmitting chimeric mice were produced by Shanghai Model Organisms Center Inc. (Shanghai, China). The Col2a1-cre mouse line was a generous gift from Dr Xiao Yang (Academy of Military Medical Sciences, Beijing, China), and Col2a1-CreERT2 mice (Stock Number: 006774) were obtained from Jackson Laboratories (Bar Harbor, ME, USA). All mice were kept on the C57BL/6J background and maintained in a standard, specific-pathogen-free facility of the Laboratory Animal Research Center of Southern Medical University. Col2a1-Cre; Myl3flox/flox mice were designated Myl3-KO, Col2a1-CreERT2; Myl3flox/flox mice were designated

Myl3-iKO and control littermates (Myl3flox/flox) were referred to as controls. Twelve-week-old male transgenic mice were used for the experimental OA and 6-, 15- and 18-month-old male transgenic mice were used for aged-OA studies. All C57BL/6J and transgenic mice were housed in a specific-pathogen-free (SPF)-level animal room with five or fewer mice per cage. Mice had free access to food and water. All mice were maintained under circulating air, constant temperature (20-26?) and a humidity of 50%-60% with a 12-hour light/dark cycle. Myl3-iKO mice and littermate control mice were intraperitoneally injected with 0.1 mg/g body weight tamoxifen (Sigma-Aldrich, CAS # 10540-29-1) dissolved in corn oil for 5 consecutive days at 11 weeks of age, and were subjected to DMM surgery at 12 weeks of age. The knees were harvested at 6 or 10 weeks after DMM surgery or at 3 or 15 months after the first injection, and subjected to histological and biochemical analyses.

Wild animals

The study did not involve wild animals

Reporting on sex

The spontaneous osteoporosis which occurs in old female mice could affect the subchondral bone, which is also an important characteristic in OA pathology. To avoid this, we use male mice in our study, according other similar studies in chondrocyte senescence. To generate male Col2a1-CreERT2; Myl3flox/flox mice, male Myl3flox/flox mice were mated with female Col2a1-CreERT2 mice to produce Col2a1-CreERT2; Myl3flox/+mice,which were then mated with Myl3flox/flox mice. The number of mice used was provided in the figure legends.

Field-collected samples

This study does not include field-collected samples.

Ethics oversight

All experimental procedures involving mice were approved by The Southern Medical University Animal Care and Use Committee.

Note that full information on the approval of the study protocol must also be provided in the manuscript.
